# Appetite-regulating hormones in early life and relationships with type of feeding and body composition in healthy term infants

**DOI:** 10.1007/s00394-016-1219-8

**Published:** 2016-05-11

**Authors:** Laura M. Breij, Monique T. Mulder, Leonie C. van Vark-van der Zee, Anita C. S. Hokken-Koelega

**Affiliations:** 1 Department of Pediatrics, Subdivision of Endocrinology, Erasmus Medical Center/Sophia Children’s Hospital, Room Number: Sk-0152, Dr. Molenwaterplein 40, 3015 GJ Rotterdam, The Netherlands; 2 Section of Pharmacology Vascular and Metabolic Diseases, Department of Internal Medicine, Cardiovascular Research School COEUR, Erasmus Medical Center, Rotterdam, The Netherlands

**Keywords:** Appetite-regulating hormones, Early life, Formula fed, Breastfed, Body composition

## Abstract

**Introduction:**

Body composition in early life influences development of obesity during childhood and beyond. Appetite-regulating hormones (ARH) play a role in regulation of food intake and might thus influence body composition in later life. Studies on associations between ARH and body composition in early life are limited.

**Methods:**

In 197 healthy term infants, we measured serum fasting levels of ghrelin, leptin, insulin, glucose-dependent insulinotropic peptide (GIP), pancreatic polypeptide (PP) and peptide YY (PYY) at 3 months and in 41 infants also at 6 months and their associations with type of feeding and longitudinal fat mass percentage (FM%) measured by air displacement plethysmography at 1, 3 and 6 months and abdominal visceral and subcutaneous fat, measured by ultrasound, at 3 and 6 months.

**Results:**

Infants with formula feeding for 3 months had significantly higher serum levels of ghrelin, leptin, insulin, GIP and PP (*p* = 0.026, *p* = 0.018, *p* = 0.002, *p* < 0.001, resp.) and lower serum levels of PYY (*p* = 0.002) at 3 months than breastfed infants. Leptin and ghrelin correlated positively with FM% at 3 months and insulin with change in FM% between 1 and 3 months (*r* = 0.40, *p* < 0.001, *r* = 0.23, *p* < 0.05, *r* = 0.22, *p* < 0.01, resp.). Leptin at 3 months correlated with subcutaneous fat at 3 months (*r* = 0.23, *p* < 0.001), but not with visceral fat. Other ARH did not correlate with body composition.

**Conclusion:**

Formula-fed infants had a different profile of ARH than breastfed infants, suggesting that lower levels of ghrelin, leptin and insulin in breastfed infants contribute to the protective role of breastfeeding against obesity development. Leptin, ghrelin and insulin were associated with fat mass percentage or its changes.

## Introduction

Childhood obesity is a worldwide problem with an increasing prevalence of 7–11 % in Dutch children aged 4–12 years [1]. It is not only associated with short-term morbidity, but also associated with long-term morbidity, such as adult obesity, type 2 diabetes and cardiovascular diseases [2–4]. Accelerated weight gain during the first 3 months of life is associated with accumulation of fat mass during childhood and a worse cardiovascular and metabolic profile in young adulthood [5–7]. Appetite-regulating hormones play a role in the regulation of food intake and body composition by signaling satiety and energy reserves through hypothalamic receptors [8]. However, little is known about the relation between body composition and appetite-regulating hormones in early life [9].

Changes in body composition might be influenced by programming of the orexigenic and anorectic appetite-regulating hormones [10]. Orexigenic appetite stimulating hormones, such as ghrelin, are important in the initiation, cessation and frequency of eating. Anorectic appetite-regulating hormones, such as leptin and peptide YY (PYY), decrease food intake and increase metabolic rate. The glucose-dependent insulinotropic peptide (GIP) stimulates pancreatic beta cells in response to the ingestion of meals or glucose [11, 12]. All these signals act at several sites in the central nervous system (CNS), but the pathways converge to the hypothalamus, which contains a large number of peptides and other neurotransmitters that influence food intake.

The protective role of breastfeeding for obesity could be partly explained by the composition of the human milk, but probably also by different appetite-regulating hormones in infants due to breastfeeding. Knowledge about the changes in appetite-regulating hormones during infancy is very limited [13].

We hypothesized that formula-fed infants would have a different profile of serum appetite-regulating hormones than breastfed infants and that fasting serum ghrelin, leptin and insulin levels would be positively associated with gain in weight and in fat mass during the first 3 months after birth, while GIP, PP and PYY levels would be negatively associated. We, therefore, investigated fasting serum levels of ghrelin, leptin and insulin, GIP, PP and PYY at 3 months and associated these with type of feeding and body composition, including FM% and visceral and subcutaneous fat at 3 and 6 months.

## Materials and methods

### Subjects

The current study is part of a birth cohort study (Sophia Pluto Study) which started in January 2013, aiming to provide detailed data on body composition and growth in early life.

Serum levels of appetite-regulating hormones (ghrelin, leptin, insulin, GIP, PYY and PP) were determined in a random subgroup of 197 infants at 3 months of age and in 41 of them also at 6 months of age.

Infants were recruited from several hospitals in and near Rotterdam, a large city in The Netherlands. All participants fulfilled the same inclusion criteria: (1) born term (≥37 weeks of gestation), (2) age <28 days and (3) uncomplicated neonatal period without signs of severe asphyxia (defined as an Apgar score below three after 5 min), sepsis or long-term complication of respiratory ventilation. Exclusion criteria were known congenital or postnatal diseases that could interfere with body composition development, confirmed intrauterine infection, maternal use of corticosteroids or significant maternal medical condition that could interfere with infant’s body composition development (e.g., diabetes).

The Medical Ethics Committee of Erasmus Medical Center approved the study. Written informed consent was obtained from both parents unless mother was single.

### Data collection and measurements

Information about the type of feeding was recorded during outpatient clinic visits.

#### Infant characteristics

Research clinic visits were scheduled at 1, 3 and 6 months. Birth data were taken from midwife and hospital records. Trained pediatric nurses took the measurements according to standard procedures.

*Anthropometrics* Weight was measured to the nearest gram by an electronic infant scale (Seca, Hanover, MD). Length was measured to the nearest 0.1 cm by a length meter (Seca). Head circumference was measured to the nearest 0.1 cm using measuring tape (Seca, circumeter). Weight SDS, height SDS and weight for length SDS were calculated with Growth Analyser Research Calculation Tools 4.0 (available at www.growthanalyser.org), according to Dutch age- and gender-matched reference values [14].

*Body composition* Whole-body composition was assessed using air displacement plethysmography (Peapod, Infant Body Composition System, COSMED). A detailed description of the air displacement plethysmography (ADP) system is provided elsewhere [15–18]. Briefly, this ADP system assesses fat mass (FM), fat mass percentage (FM%) and fat-free mass (FFM) and fat-free mass percentage (FFM%) by direct measurements of body volume and body mass, based on the whole-body densitometric principle. All measurements were obtained by experienced personnel, according to standardized protocol. The Peapod was calibrated every day, according to the protocol recommended by the supplier.

*Abdominal fat* Visceral and abdominal subcutaneous fat was measured at 3 and 6 months using a Prosound 2 ultrasound, with a UST-9137 convex ultrasound transducer (both from Hitachi Aloka Medical, Switzerland). For both measures, the transducer was positioned where the xiphoid line intercepted the waist circumference measurement plane. Visceral fat was estimated by measuring visceral depth, which is the distance between the peritoneal boundary and the corpus of the lumbar vertebra, assessed in the longitudinal plane with the ultrasound probe depth set at 9 cm. Subcutaneous abdominal fat was estimated by the distance between the cutaneous boundary and the linea alba at the same location, but on a transverse plane with a probe depth of 4 cm [19].

### Collection of blood and assays

At 3 months, blood samples were collected from a heel prick after the infants had fasted for at least 3 h. To stabilize the appetite-regulating hormones, blood samples were collected in EDTA tubes and dipeptidyl peptidase-4 inhibitor (DPP4-inhibitor, Merck Chemicals) and 4-(2-aminoethyl)benzenesulfonyl fluoride hydrochloride (serine protease inhibitor, Calbiochem) were added at the time of collection. Blood was centrifuged at 4 °C to prepare plasma, which was quickly frozen. Samples were stored at −80 °C.

Ghrelin (active), leptin, insulin, GIP, PP and PYY concentrations in serum were determined by the MILLIPLEX MAP Human Metabolic Hormone Magnetic Bead Panel, catalog number HMHMAG-34K (Millipore Corporation, Billerica, MA) using the commercial protocol. The intra-assay CV was <10 %, and the inter-assay CV was <15 %.

### Statistical analysis

Descriptive results are expressed as median (interquartile range). Differences between groups were examined using Mann–Whitney *U* tests. We assessed linear correlations between levels of ghrelin, leptin, insulin, GIP, PP and PYY and other parameters using Pearson’s correlation coefficient. Correlation coefficient below 0.20 was considered to be a negligible correlation. SPSS statistical package version 20.0 (SPSS Inc. Chicago, Illinois) was used. All statistical tests were performed two-sided, and results were regarded statistically significant if the *P* value was <0.05.

## Results

Clinical characteristics of the infants are presented in Table 1. The median (IQR) gestational age was 39.9 (38.9–40.6) weeks, and 53 % of the infants were boys. Forty-one boys had their appetite-regulating hormones longitudinally determined at 3 and 6 months. Fifty-six percentage of the infants received exclusive breastfeeding at 1 month, 32 % at 3 months, and 16 % at 6 months.

**Table 1:** Clinical characteristics

	Total group		Girls (*n* = 93)	Boys (*n* = 104)	*p* value♀ versus ♂	Subgroup (*n* = 41)	*p* value^#^
	(*n* = 197)						
*Birth*							
Gestational age (weeks)	39.9	38.9 to 40.6	39.9	39.7	0.58	39.4	**0.03**
Birth weight SDS	−0.38	−1.12 to 0.33	−0.42	−0.35	0.60	−0.85	0.08
Birth length SDS	0.13	−1.05 to 0.80	0.13	−0.38	0.47	−0.97	0.10
Age 1 month							
Age (months)	0.95	0.92 to 1.05	0.95	0.99	0.38	0.95	0.34
Weight SDS	0.33	−0.51 to 1.09	0.42	0.33	0.79	−0.04	0.09
Length SDS	0.04	−0.61 to 0.68	0.04	0.02	0.61	−0.11	**0.03**
Sum of peripheral skinfolds (mm)	11.0	10.0 to 13.0	11.0	11.0	0.73	11.0	0.33
Sum of central skinfolds (mm)	11.0	9.0 to 12.0	11.0	11.0	0.67	11.0	0.46
Fat mass (kg)	0.68	0.54 to 0.86	0.65	0.73	0.62	0.74	0.65
Fat mass percentage (%)	16.4	13.7 to 19.4	16.5	16.3	0.39	17.7	0.49
Age 3 months							
Age (months)	2.99	2.92 to 3.06	2.99	2.99	0.90	2.99	0.40
Weight SDS	0.49	−0.24 to 1.22	0.36	0.57	**0.05**	0.27	0.58
Length SDS	0.43	−0.17 to 0.90	0.27	0.56	**0.04**	0.18	0.14
Sum of peripheral skinfolds (mm)	15.0	13.0 to 16.0	14.0	15.0	0.10	15.0	0.28
Sum of central skinfolds (mm)	13.0	11.0 to 15.0	12.0	13.0	0.53	13.0	**0.05**
Fat mass (kg)	1.33	1.12 to 1.59	1.29	1.37	0.23	1.32	0.79
Fat mass percentage (%)	22.6	19.7 to 25.8	23.1	22.4	0.34	23.2	0.92
Visceral fat (cm)	2.53	2.03 to 2.89	2.39	2.54	0.23	2.54	0.31
Abdominal subcutaneous fat (cm)	0.42	0.35 to 0.50	0.42	0.43	0.14	0.41	0.42
Age 6 months							
Age (months)	6.01	5.95 to 6.11	6.01	6.01	0.57	5.98	0.57
Weight SDS	0.14	−0.31 to 0.71	0.06	0.18	0.33	0.09	0.37
Length SDS	0.29	−0.34 to 0.78	0.25	0.31	0.64	0.20	0.14
Sum of peripheral skinfolds (mm)	16.0	14.0 to 17.0	17.0	15.0	0.18	15.0	0.75
Sum of central skinfolds (mm)	12.0	11.0 to 15.0	13.0	12.0	0.36	13.0	0.85
Fat mass (kg)	1.76	1.44 to 2.09	1.79	1.75	0.42	1.70	0.31
Fat mass percentage (%)	23.5	20.2 to 27.3	24.8	22.8	**<0.01**	22.6	0.16
Visceral fat (cm)	2.34	2.00 to 2.84	2.34	2.45	0.80	2.19	0.91
Abdominal subcutaneous fat (cm)	0.41	0.35 to 0.51	0.45	0.41	0.63	0.40	0.39

Data expressed as median (interquartile range)

Significant *P* values are indicated in boldface. # Differences between total group and subgroup

Table 2 shows serum levels of ghrelin, leptin, insulin, GIP, PP and PYY at 3 and 6 months. Median fasting time had been 3.00 (2:30–3:40) hours at 3 months and 2.52 (2:13–4:15) hours at 6 months. Ghrelin, GIP and PP levels increased significantly from 3 to 6 months (*p* < 0.001, *p* = 0.016, *p* < 0.001, resp.), while the leptin levels decreased between 3 and 6 months (*p* = 0.009).

**Table 2:** Serum levels of appetite-regulating hormones in infants at 3 and 6 months of age

	3 months				6 months		*p* value girls vs. boys at 3 months
	Girls		Boys		Boys		
Total group (*n* = 197)							
Hours fasting (h)	2:50	2:22–3:30	3:12	2:30–3:55			0.11
Ghrelin (pg/ml)	44.0	23.4–82.1	48.9	23.4–68.3			0.57
Leptin (pg/ml)	1439.6	875.3–2364.7	1405.2	742.6–2001.4			0.24
Insulin (pg/ml)	369.0	232.3–619.5	430.5	265.7–738.1			0.30
GIP (pg/ml)	244.8	133.0–393.1	237.3	138.5–350.8			0.87
PP (pg/ml)	59.8	31.7–94.2	68.3	46.5–104.1			0.08
PYY (pg/ml)	210.6	159.4–293.8	196.9	156.3–250.8			0.24

							*p* value 3 versus 6 months
*Subgroup (n* *=* *41)*							
Hours fasting (h)			3:25	2:38–4:00	2.52	2.13–4.15	0.252
Ghrelin (pg/ml)			46.2	20.2–67.6	83.1	48.7–102.6	**<0.001**
Leptin (pg/ml)			1560.4	945.4–1991.4	827.2	424.6–1247.2	**<0.001**
Insulin (pg/ml)			464.9	291.6–718.5	483.8	324.4–635.6	0.821
GIP (pg/ml)			263.1	137.1–345.4	310.9	201.5–421.1	**0.008**
PP (pg/ml)			70.9	41.4–127.8	115.7	68.4–241.6	**<0.001**
PYY (pg/ml)			171.6	150.1–231.7	158.7	130.7–205.6	0.065

Data expressed as median (interquartile range)

Significant *P* values are indicated in boldface

There were no differences in appetite-regulating hormone levels between boys and girls (Table 2).

### Associations between appetite-regulating hormones and type of feeding

Table 3 shows the serum levels of appetite-regulating hormones during exclusively formula feeding versus exclusively breastfeeding for 3 months. There were no significant differences in weight SDS, FM%, visceral and subcutaneous fat at 1, 3 and 6 months between the formula-fed and breastfed infants, but all appetite-regulating hormones were different between the formula-fed and breastfed groups. Serum levels of ghrelin, leptin, insulin, GIP and PP were significantly higher in the formula-fed group compared to the breastfed group (*p* = 0.026, *p* = 0.018, *p* = 0.002, *p* < 0.001, resp.), whereas PYY was significantly lower (*p* = 0.002).

**Table 3:** Serum levels of appetite-regulating hormones in 197 infants at 3 months, divided by type of feeding

	Exclusive FF at 3 months	Exclusive BF at 3 months	*p* value
Ghrelin (pg/ml)	49.1	33.2	**0.026**
Leptin (pg/ml)	1719.1	1190.1	**0.018**
Insulin (pg/ml)	560.9	287.8	**<0.001**
GIP (pg/ml)	304.1	198.5	**0.002**
PP (pg/ml)	87.7	46.3	**<0.001**
PYY (pg/ml)	180.2	231.0	**0.002**

Data were expressed as medians. Significant *P* values are indicated in boldface

At 3 months, a shorter duration of exclusive breastfeeding correlated only with a higher PP level (*r* = −0.24, *p* = 0.005). At 6 months, we found no significant correlations of duration of breastfeeding with appetite-regulating hormones.

### Associations between appetite-regulating hormones and fat mass percentage

Serum leptin at 3 months correlated with fat mass percentage (FM%) at that age (*r* = 0.37, *p* < 0.001) (Table 4). Similar associations were found between serum leptin at 3 months and FM% at 6 months (*r* = 0.41, *p* < 0.001). Serum insulin at 3 months correlated with the increase in FM% between 1 and 3 months (*r* = 0.22, *p* < 0.01). The other appetite-regulating hormones did neither correlate with FM% nor correlate with changes in FM%. We found in the formula-fed infants a positive correlation between ghrelin at 3 months and FM% (*r* = 0.23, *p* < 0.05) at that age. In the breastfed infants, we did not find this correlation, but a stronger correlation between leptin and FM% (*r* = 0.61, *p* < 0.001) (data not shown).

**Table 4:** Correlations of appetite-regulating hormones with body composition and anthropometry at 3 months in 197 infants

	FM%	Delta FM% 1–3 months	Visceral fat	Subcutaneous fat	Weight for length SDS	Delta weight for length SDS 1–3 months
Ghrelin (pg/ml)	0.11	0.01	<0.01	<0.01	0.06	0.04
Leptin (pg/ml)	0.37**	0.04	0.01	0.16^	0.48**	0.45**
Insulin (pg/ml)	<0.01	0.22*	0.19*	−0.01	0.08	0.20*
GIP (pg/ml)	0.03	−0.16^	0.11	−0.08	0.04	0.13
PP (pg/ml)	0.04	−0.02	0.04	−0.02	0.03	0.09
PYY (pg/ml)	−0.08	0.03	0.03	−0.12	−0.23*	−0.12

Significant *P* values are indicated in boldface if *r* > 0.20 and *p* < 0.05; ^ *p* < 0.05; * *p *< 0.01; ** *p* <  0.001

### Associations between appetite-regulating hormones and visceral and subcutaneous fat

Serum leptin, ghrelin and insulin at 3 months did not correlate with visceral and subcutaneous fat at 3 and 6 months. Breastfed infants showed a correlation between leptin and subcutaneous fat at 3 months (*r* = 0.38, *p* < 0.01) (data not shown).

### Associations between appetite-regulating hormones and anthropometrics

As a proxy for body composition, anthropometrics are often used. We, therefore, also analyzed correlations between appetite-regulating hormones and anthropometrics. Serum leptin at 3 months correlated positively with weight for length SDS at 3 months (*r* = 0.48, *p* < 0.001) and 6 months (data not shown) (Table 4). A higher PYY at 3 months correlated with lower weight for length SDS at 3 months (*r* = −0.23, *p* < 0.01). Serum leptin and insulin at 3 months correlated with the increase in weight for length SDS between 1 and 3 months of life (*r* = 0.45, *p* < 0.001, *r* = 0.20, *p* < 0.01, resp.).

## Discussion

In this study, we investigated the fasting serum levels of appetite-regulating hormones, such as ghrelin, leptin, insulin, GIP, PP and PYY in infants at 3 and 6 months and their associations with type of feeding, FM%, visceral fat and subcutaneous fat. Interestingly, all appetite-regulating hormones were different between infants with formula feeding compared to the breastfed infants. Serum levels of ghrelin, leptin, insulin, GIP and PP were significantly higher in the formula-fed group, whereas PYY was significantly lower. Leptin at 3 months correlated positively with FM% at 3 and 6 months, but only with subcutaneous fat and not with visceral fat. Also ghrelin at 3 months correlated with FM% at 3 months, but only in formula-fed infants. Serum insulin correlated positively with gain in FM% between 1 and 3 months. Other appetite-regulating hormones were not associated with fat mass percentage and visceral fat in the first 3–6 months.

We show that formula-fed infants have different levels of appetite-regulating hormones than infants with exclusive breastfeeding. For healthy term infants, exclusive breastfeeding is considered the reference to which formula feeding must be compared. Serum levels of ghrelin were significantly higher in formula fed than in breastfed infants. These data are in line with a cross-sectional study where the same results were found in the first year of life in a group of Italian infants [20]. It has been demonstrated in animal studies that neonatal ghrelin is important for normal maturation of hypothalamic neural circuits. The neural developmental activity of ghrelin is essentially restricted to the neonatal period [21]. Proper expression of ghrelin during neonatal life is, therefore, crucial for lifelong metabolic regulation, and too high ghrelin levels during the first months of life result in lifelong metabolic disturbances [22]. In the formula-fed infants, we did not find very high levels of ghrelin, but their levels were significantly higher than in the breastfed infants. In this critical period, this could have adverse effects on the maturation of the hypothalamic neural circuits, resulting in less favorable metabolic regulation in later life [23]. We found also higher leptin levels in formula-fed infants. Like ghrelin, leptin is known to promote the development of the arcuate nucleus in the hypothalamus, and its developmental action is also restricted to a critical window in the first months of life [24], but higher ghrelin levels could impair leptin signaling in the arcuate nucleus in early life [21]. Our data are in line with another study, reporting higher leptin levels in formula-fed infants compared to breastfed infants [25]. However, in a study in infants with a smaller group of subjects, breastfed infants had higher leptin levels than formula-fed infants [26].

Infants with formula feeding had lower levels of PYY. One of the actions of PYY is to reduce food intake by reducing the gastro-intestinal tract motility and the gastric emptying, via the vagal–brainstem–hypothalamic pathway [27]. As the breastfed infants had higher PYY levels, that could be a link in the protective role of breastfeeding for obesity. In addition, we found that higher levels of PYY were associated with a lower weight for length SDS at 3 months. These results are in line with the data of Helsinki Birth Cohort Study, where they found higher PYY levels in adulthood when the infants had a lower growth rate during infancy [28]. Our study shows that these differences can already be found in early life. Infants with formula feeding had also higher levels of insulin, GIP and PP. A possible explanation of the higher insulin levels in formula-fed infants could be the amount of proteins, which is in general higher in formula feeding than in breastfeeding [29]. Another higher appetite-regulating hormone in formula-fed infants was GIP, which plays an important role in the regulation of plasma glucose and insulin secretion [30]. It is possible that higher GIP levels could account for an enhanced insulin release [31]. Also PP was higher in formula-fed infants. The function of PP is to self-regulate pancreatic secretion activities [32]. However, the mechanism of how the type of feeding influences the GIP and PP levels is not fully understood. For further interpretation of the potential relevance of these findings, more research is required.

To our knowledge, this is the first large study investigating the relationship between simultaneously measured serum levels of appetite-regulating hormones and detailed measurements of infant body composition, by air displacement plethysmography (Peapod). We found a significant positive correlation between serum ghrelin and FM% at 3 months in the formula-fed infants, but not in the breastfed group. Leptin was also correlated with FM% in both formula-fed and breastfed groups. The associations of leptin with FM% could be explained by the known physiology of adipose tissue. During early life, the adipocytes proliferate and differentiate [33]. Leptin levels increase in parallel to the number of adipocytes during the last part of the third trimester during gestation and the first months of life [34]. We demonstrated that leptin levels in male infants were higher at 3 months than at 6 months. Serum insulin correlated positively with gain in FM% between 1 and 3 months. Earlier studies showed a positive association of insulin and insulin resistance with fat mass in childhood and adulthood [35, 36]. The other appetite-regulating hormones such as PP and GIP did not associate with FM% or gain in FM% in the total group, which partly contrasted our expectations. There is a need for further studies to identify the underlying mechanisms and potential implications for possible programming effects of serum appetite hormones on later body composition.

We could not identify differences in appetite-regulating hormones between boys and girls, which is in line with studies in older children and in a study with a subset of appetite-regulating hormones in infants [30, 37, 38].

We did not investigate hormone levels in breast milk. Some studies investigated levels of ghrelin, leptin or insulin in breast milk [9, 39–42]. In these studies, associations between appetite-regulating hormones in breast milk and anthropometric data of infants were found. An unhealthy maternal diet can lead to an unfavorable appetite-regulating hormone profile in breast milk, but it is not clear by which mechanism this unfavorable profile in breast milk could influence infant growth as hormones will be degraded when passing the stomach. In our study, we only investigated fasting levels of appetite-regulating hormones. We realize that several appetite-regulating hormones are low in fasting state [30], but our study shows that even in fasting state all hormones were different between the formula-fed and breastfed infants. It would be interesting to investigate appetite-regulating hormone levels before and after a meal, but in our study, it was found unethical to collect blood for research purpose twice in healthy infants.

In conclusion, we found significantly higher serum levels of ghrelin, leptin, insulin, GIP and PP and lower serum levels of PYY in infants who received exclusive formula feeding for at least 3 months compared to breastfeeding. Besides associations of leptin, ghrelin and insulin with body composition in infants of 3 and 6 months of age, we could not identify other associations of serum appetite-regulating hormones with body composition. Further studies on appetite-regulating hormones and the interactions between these and body composition later in infancy would be helpful to further elucidate whether appetite-regulating hormones contribute to the programming of metabolic health in later life.
